# Bidirectional lysosome transport: a balancing act between ARL8 effectors

**DOI:** 10.1038/s41467-022-32965-y

**Published:** 2022-09-07

**Authors:** Agnieszka A. Kendrick, Jenna R. Christensen

**Affiliations:** Department of Cellular and Molecular Medicine, University of California, San Diego, La Jolla, CA 92093 USA

**Keywords:** Motor proteins, Lysosomes

## Abstract

Most organelles move bidirectionally on microtubule tracks, yet how this opposing movement is regulated by kinesin and dynein remains unclear. Recent work found that ARL8, a known anterograde adaptor linking the lysosome to kinesin, also links lysosomes to the retrograde motor dynein, providing key insight into bidirectional organelle movement in cells.

## Regulation of bidirectional lysosome movement

Cellular cargos achieve precise spatial and temporal distribution via bidirectional transport on microtubule tracks. One bidirectionally-transported cargo is the lysosome. Lysosomes (here, the term ‘lysosome’ collectively refers to late endosomes, lysosomes, and endolysosomes) are membrane-enclosed structures crucial for cellular health and energy homeostasis. Lysosomes undergo ‘retrograde’ transport toward microtubule minus-ends at the centrosome via cytoplasmic dynein-1 (dynein here) and ‘anterograde’ transport toward microtubule plus-ends at the cell periphery via kinesin motors. Environmental cues, such as nutrient availability, viral infection, and cellular stress regulate the localization and corresponding functions of lysosomes^1^. For example, nutrient withdrawal leads to lysosome retrograde transport and perinuclear clustering^2^. There, lysosomes fuse with autophagosomes, which in turn shuts off lysosome-mediated mTORC1 signaling and activates autophagy. In contrast, the re-introduction of growth factors triggers anterograde lysosome movement toward the cell periphery and re-activates mTORC1 signaling required for protein synthesis and inhibition of autophagy^3^. Nutrient-driven lysosome positioning is also important for ER remodeling and focal adhesion disassembly at the cell membrane^4,5^. Thus, bidirectional lysosome movement and distribution are key aspects of cellular adaptation to environmental cues. However, the precise molecular mechanisms linking lysosomes to the anterograde or retrograde transport systems are not fully understood.

## ARL8 links lysosomes to kinesin for anterograde transport

The opposite-polarity motors dynein and kinesin often associate with cellular organelles via adaptor proteins that link to a small GTPase of the Rab, Arf, or Arf-like (Arl) families^6^. GTPases cycle through inactive GDP-bound and active, membrane-associated GTP-bound forms. GTPase “effector” proteins directly bind to GTPases in their membrane-associated, GTP-bound form. These effectors can link GTPases to opposite-polarity motors, resulting in organelle movement. Lysosomes are one prime example where GTPases regulate organelle transport in both the anterograde and retrograde directions. For example, the Rab7 GTPase links lysosomes to dynein-dynactin via the RILP adaptor, and to kinesin via FYCO1^7–9^.

**Fig. 1:**
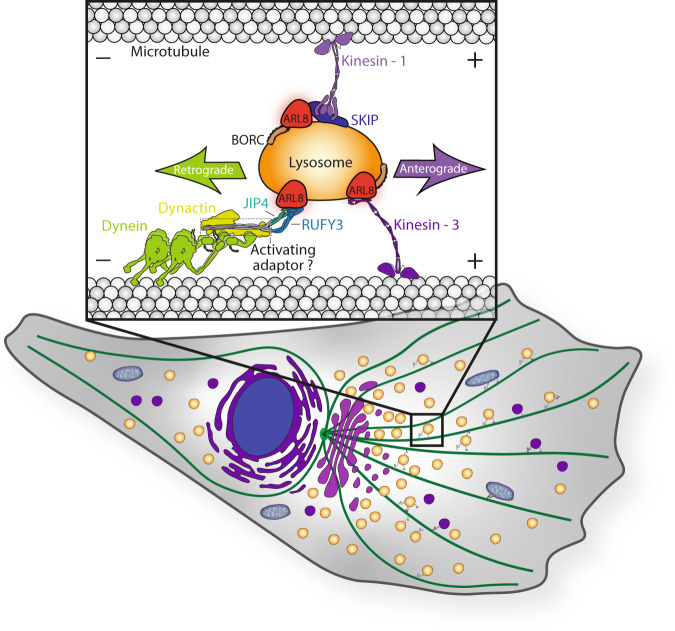
Bidirectional lysosome movement is mediated by ARL8. Lysosomes (orange) are transported in the anterograde direction towards the cell periphery or in the retrograde direction toward the cell center on microtubule tracks (green). Inset: ARL8 (red) interacts with the BORC complex and links lysosomes directly to kinesin-3 or to kinesin-1 via SKIP for anterograde transport. ARL8 interacts with RUFY3 to link lysosomes to the dynein-dynactin complex for retrograde transport either directly or via the JIP4 adaptor. Dynein requires activation by dynactin and an activating adaptor. Both JIP4 and RUFY3 share similarities to other dynein activating adaptors, suggesting they could potentially activate the dynein complex for motility (dotted line).

Another well-established GTPase that regulates lysosome transport is ARL8. Humans have two ARL8 paralogs, ARL8A and ARL8B, with overlapping functions. ARL8 has been demonstrated to associate with kinesins both directly and indirectly, driving lysosome transport in the anterograde direction (Fig. 1). ARL8 directly recruits kinesin-3 to lysosomes and indirectly associates with kinesin-1 by binding to the adaptor protein SKIP (also known as PLEKHM2)^10,11^. Recruitment of different kinesins by these distinct mechanisms can then facilitate lysosome transport on microtubules with diverse post-translational modifications^12^. Though ARL8 has a key role in lysosome transport in the anterograde direction, up until now, ARL8 has not been linked to retrograde lysosome transport.

## Two recent studies link ARL8 to dynein-mediated transport

Two recent studies by Keren-Kaplan et al.^13^ and Kumar et al.^14^ shed light on how ARL8 also acts as a regulator of lysosome retrograde transport. Keren-Kaplan et al. and Kumar et al. identify the RUN- and FYVE-domain-containing protein RUFY3 as a novel ARL8 effector that links lysosomes to the dynein complex for retrograde transport (Fig. 1). Their findings provide a novel example of how small GTPases such as ARL8 regulate the bidirectional movement of cellular structures by linking opposite-polarity motors to cellular cargo.

In these two studies, RUFY3 was identified as an ARL8 interactor by different experimental techniques. Keren-Kaplan et al. used MitoID, a proteomics approach in which the bait protein, ARL8 in this case, is associated with a promiscuous biotin ligase and targeted to mitochondria. This technique identified the ARL8 ‘interactome’ while reducing non-specific cellular interactions. Concurrently, Kumar et al. performed a yeast-two-hybrid experiment with ARL8B and a human brain tissue cDNA library. Both groups identified RUFY3 as an ARL8 interactor and used co-immunoprecipitation and cellular co-localization assays to confirm that RUFY3 associates with GTP-bound ARL8, suggesting that RUFY3 is an ARL8 effector. In addition, Keren-Kaplan et al. showed using co-immunoprecipitation and co-localization studies that RUFY4, but not other members of the RUFY family, can also associate with ARL8. The ARL8-interaction region within RUFY3 was mapped to its coiled-coil (CC2) and FYVE domains. Kumar and colleagues further mapped this interaction to residues R462/K465 and showed using purified proteins that the interaction between RUFY3 and ARL8 is direct, confirming RUFY3 as a novel ARL8 effector.

Multiple studies have shown that ARL8 is critical for kinesin-mediated lysosome movement toward the cell periphery (reviewed in ref. 15). However, overexpression of RUFY3 or RUFY4 (Keren-Kaplan et al.) or co-overexpression of RUFY3 and ARL8 (Kumar et al.) led to a clustering of lysosomes at the nucleus, indicating retrograde, or dynein-mediated, lysosome transport. Therefore, both groups tested if RUFY proteins associate with components of the dynein complex. To achieve processive motility, dynein requires association with the protein complex dynactin and an activating adaptor protein that also links the complex to cargos^16–19^. Keren-Kaplan et al. showed that RUFY3 and RUFY4 associate with dynein-dynactin complex components via immunoprecipitation, while Kumar et al. performed affinity purification mass spectrometry of GST-RUFY3 in HEK293T cell lysates and identified dynein and dynactin subunits.

In follow-up experiments, both groups sought to determine how RUFY proteins mediate the association of ARL8 with dynein-dynactin. Keren-Kaplan et al. found that RUFY3 directly interacted with the C-terminal region of the dynein subunit DLIC1, a mode of interaction similar to numerous dynein activating adaptors^20^. On the other hand, experiments by Kumar et al. demonstrate that RUFY3 may indirectly associate with dynein. Mass spectrometry of RUFY3 identified the proposed dynein activating adaptor JIP4, and JIP4 was required for RUFY3-mediated clustering of lysosomes at the nucleus. Collectively, these data show that RUFY proteins link ARL8-marked lysosomes to the dynein complex either directly or via a JIP4 adaptor.

Finally, both groups tested the contribution of RUFY3-dependent lysosome movement to changes in the cellular environment. During nutrient depletion, the cytoplasmic pH increases, and lysosomes move in the retrograde direction to eventually accumulate at the perinuclear region^2^. Keren-Kaplan et al. found that shifting the cytoplasm to a higher pH in RUFY3-depleted cells led to a partial reduction in lysosome perinuclear clustering. Similarly, Kumar et al. showed that RUFY3 was required for lysosome repositioning following nutrient depletion. Therefore, the cellular environment can influence the directionality of lysosome transport.

## Bidirectional transport regulation occurs at multiple levels

This work and the work of others have elucidated the multiple layers of regulation that act on bidirectional transport at the level of the organelle, the GTPase, the effector, or the motors. At the organelle level, different GTPases can be recruited to the lysosome via different mechanisms. For example, Rab7 attaches to membranes via carboxy-terminal prenyl groups, and this process is tightly controlled by Rab7 binding proteins^21^. ARL8 lacks the carboxy-terminal residues required for prenylation and instead requires acetylation in its amino-terminal region, as well as association with the BORC complex^12,22^. Therefore, the amount or type of each GTPase and/or regulatory proteins present on a specific cargo, as well as their rate of GTP hydrolysis could define organelle movement at a broad level.

At the GTPase level, a single GTPase is capable of recruiting multiple different effectors. The ARL8 GTPase can directly recruit kinesin-3, indirectly recruit kinesin-1 via SKIP, or recruit dynein-dynactin via RUFY3 or RUFY4, promoting movement on distinct microtubule tracks or in different directions^11–14^. Future work will unravel if these effectors compete for ARL8 binding and what factors impact effector binding.

At the effector level, distinct effectors may recruit the same motor by different mechanisms. The ARL8 effector RUFY3 may directly recruit dynein-dynactin via an interaction with the DLIC, or indirectly via JIP4. Given that dynein requires an association with dynactin and activating adaptor to achieve motility, it remains to be determined if RUFY3 and/or JIP4 are sufficient to activate dynein for motility in addition to linking it to lysosomes. A recent preprint shows the recruitment of tandem activating adaptors to dynein-dynactin, raising the possibility of a potential mechanism where RUFY3 and JIP4 could engage the dynein complex at the same time or utilize another dynein activating adaptor for dynein-mediated motility^23^. It is possible that recruiting or activating dynein-dynactin by these different mechanisms may result in retrograde movement that differs in speed, run length, or other characteristics.

Finally, at the motor level, different motors may compete for microtubule engagement. Cargos are likely bound by many motors at once. Opposite-polarity motors may engage in a ‘tug-of-war’ with each other, resulting in cargo movement in the direction of the ‘winning’ set of motors. The number of motors can also influence the run length or speed of moving lysosomes, providing an additional level of regulation. It is also possible that the cellular environment, and factors like nutrient depletion, may influence transport regulation at these different levels. Clearly, there are multiple layers of regulation that fine-tune the transport of cargo under different cellular circumstances. Lysosomes will continue to present an ideal test case for understanding the diverse aspects of bidirectional cargo transport in different environmental contexts.

## References

[1] Raiborg C (2018). How nutrients orchestrate lysosome positioning. Contact.

[2] Korolchuk VI (2011). Lysosomal positioning coordinates cellular nutrient responses. Nat. Cell Biol..

[3] Jia R, Bonifacino JS (2019). Lysosome positioning influences mTORC2 and AKT signaling. Mol. Cell..

[4] Lu, M. et al. The structure and global distribution of the endoplasmic reticulum network are actively regulated by lysosomes. *Sci. Adv*. **6**, eabc7209 (2020). 10.1126/sciadv.abc7209 PMC7744115 33328230

[5] Schiefermeier N (2014). The late endosomal p14-MP1 (LAMTOR2/3) complex regulates focal adhesion dynamics during cell migration. J. Cell Biol..

[6] Kjos I, Vestre K, Guadagno NA, Borg Distefano M, Progida C (2018). Rab and Arf proteins at the crossroad between membrane transport and cytoskeleton dynamics. Biochim. Biophys. Acta Mol. Cell Res..

[7] Johansson M (2007). Activation of endosomal dynein motors by stepwise assembly of Rab7-RILP-p150Glued, ORP1L, and the receptor betalll spectrin. J. Cell Biol..

[8] Pankiv S (2010). FYCO1 is a Rab7 effector that binds to LC3 and PI3P to mediate microtubule plus end-directed vesicle transport. J. Cell Biol..

[9] Raiborg C (2015). Repeated ER–endosome contacts promote endosome translocation and neurite outgrowth. Nature.

[10] Niwa S (2016). Autoinhibition of a neuronal kinesin UNC-104/KIF1A regulates the size and density of synapses. Cell Rep..

[11] Rosa-Ferreira C, Munro S (2011). Arl8 and SKIP act together to link lysosomes to kinesin-1. Dev. Cell..

[12] Guardia CM, Farías GG, Jia R, Pu J, Bonifacino JS (2016). BORC functions upstream of kinesins 1 and 3 to coordinate regional movement of lysosomes along different microtubule tracks. Cell Rep..

[13] Keren-Kaplan T (2022). RUFY3 and RUFY4 are ARL8 effectors that promote coupling of endolysosomes to dynein-dynactin. Nat. Commun..

[14] Kumar G (2022). RUFY3 links Arl8b and JIP4-dynein complex to regulate lysosome size and positioning. Nat. Commun..

[15] Khatter D, Sindhwani A, Sharma M (2015). Arf-like GTPase Arl8: moving from the periphery to the center of lysosomal biology. Cell Logist..

[16] McKenney RJ, Huynh W, Tanenbaum ME, Bhabha G, Vale RD (2014). Activation of cytoplasmic dynein motility by dynactin-cargo adapter complexes. Science.

[17] Reck-Peterson SL, Redwine WB, Vale RD, Carter AP (2018). The cytoplasmic dynein transport machinery and its many cargoes. Nat. Rev. Mol. Cell Biol..

[18] Olenick MA, Holzbaur ELF (2019). Dynein activators and adaptors at a glance. J. Cell Sci..

[19] Schlager MA, Hoang HT, Urnavicius L, Bullock SL, Carter AP (2014). In vitro reconstitution of a highly processive recombinant human dynein complex. EMBO J.

[20] Lee I-G, Cason SE, Alqassim SS, Holzbaur ELF, Dominguez R (2020). A tunable LIC1-adaptor interaction modulates dynein activity in a cargo-specific manner. Nat. Commun..

[21] Pylypenko O, Hammich H, Yu I-M, Houdusse A (2018). Rab GTPases and their interacting protein partners: structural insights into Rab functional diversity. Small GTPases..

[22] Hofmann I, Munro S (2006). An N-terminally acetylated Arf-like GTPase is localised to lysosomes and affects their motility. J. Cell Sci..

[23] Chaaban, S., & Carter, A. P. Structure of dynein-dynactin on microtubules shows tandem recruitment of cargo adaptors. Preprint at https://www.biorxiv.org/content/10.1101/2022.03.17.482250v1 (2022). 10.1038/s41586-022-05186-y PMC7613678 36071160

